# Information mastery skills among pre-clerkship students in a problem-based learning curriculum: a case report

**DOI:** 10.5195/jmla.2026.2203

**Published:** 2026-01-01

**Authors:** Christopher Duffy, Tovah Tripp, Ezra Schneier, Margaret Dreker, Miriam Hoffman, Joshua Josephs

**Affiliations:** 1 christopher.duffy@hmhn.org, Associate Dean, Medical Library, Hackensack Meridian School of Medicine, Nutley, NJ; 2 tovah.tripp@hmhn.org, Internal Medicine Clerkship Director, Hackensack University Medical Center, Department of Internal Medicine, 30 Prospect Ave, Hackensack, NJ; 3 ezra.schneier@hmsom.edu, Resident Physician, Internal Medicine, Hackensack University Medical Center, Nutley, NJ; 4 margaret.dreker@hmhn.org, Medical Librarian, Hackensack Meridian School of Medicine, Nutley, NJ; 5 miriam.hoffman@hmsom.edu, Vice Dean for Academic Affairs, Hackensack Meridian School of Medicine, Nutley, NJ; 6 joshua.josephs@hmhn.org, Director of Health Systems Science Curriculum, Hackensack University Medical Center, Department of Internal Medicine, 30 Prospect Ave, Hackensack, NJ

**Keywords:** Evidence-based medicine, problem-based learning, assessment, health systems science

## Abstract

**Background:**

Use of evidence-based medicine (EBM) can improve patient outcomes, but translating classroom learning of EBM to clinical practice is challenging. Training students to utilize and apply principles of EBM is critical but data and methods for evaluating students' EBM skills are lacking.

**Case Presentation:**

The Hackensack Meridian School of Medicine has early curricular introduction of information mastery techniques to combat these challenges. Students create research presentations related to the weekly problem-based-learning (PBL) case to practice applying EBM skills. Medical librarians developed and utilized an assessment tool to evaluate students' weekly presentations. Librarian staff reviewed 595 presentations during the first year of the pre-clerkship curriculum using five criteria: (1) appropriate scope of presentation (2) correct categorization of the question based on the finding information framework (3) appropriate resource used (4) search strategy and (5) bibliographic citations according to American Medical Association (AMA) guidelines.

**Conclusions:**

Of the evaluated presentations using these criteria, the majority of students routinely and reliably applied EBM skills in their case-based presentations. Further studies will need to look at continued development of these skills throughout other phases of training.

## BACKGROUND

Healthcare quality in the United States, despite its advanced technologies and substantial healthcare spending, continues to lag behind other developed nations in key areas such as patient outcomes, access to care, and cost effectiveness [1]. Fragmented care and inconsistent clinical practice are among the causes of these disparities [2]. Evidence-based medicine (EBM), which emphasizes integrating research evidence, clinical expertise, and patient values into clinical decision making, has the potential to address the aforementioned challenges [2,3]. While the principles of EBM have been part of medical education for over two decades [4], translating EBM knowledge into clinical practice is inconsistent [5]. Differing teaching methods, varied clinical exposures, and lack of standardized assessment all contribute to difficulties in application and translation of EBM from the classroom to clinical practice [2,4].

In the pre-clerkship setting, students primarily ask background questions—those aimed at understanding general concepts—because their limited medical knowledge as first-year students often leads them to focus on foundational topics such as physiology. Unlike clinical questions which can be asked and answered narrowly using the PICO (patient, intervention, comparison, outcome) format [6,7], no format exists for asking clinical background questions. Various methods for teaching and evaluating pre-clinical students’ evidence-based medicine (EBM) skills have been explored in the literature. Approaches such as flipped classroom models—which combine asynchronous modules with in-person teaching sessions—have been studied and shown to be effective. Early instruction and assessment of EBM skills have demonstrated measurable benefits regarding student's confidence in forming clinical questions and critically appraising medical evidence. [5,8,9] However, we have not found any studies that describe a longitudinal integration of medical librarians into a pre-clinical curriculum that teaches and assesses medical students’ EBM skills.

## CASE PRESENTATION

At the Hackensack Meridian School of Medicine (HMSOM), we sought to bridge the gap between EBM knowledge, application and evaluation using our modified Problem-Based Learning (PBL) curriculum, Patient Presentation Problem-Based-Learning Curriculum (PPPC)[10]. This longitudinal course spans the entirety of the pre-clerkship curriculum and is integrated with the basic science and systems courses.

Each week, students are presented with a case that integrates basic, clinical and health systems science. Students engage in a self-directed learning process related to the clinical case that requires them to identify a knowledge gap, create a research question, and then use appropriate resources to research and present the answer to that question. Utilizing the Finding Information Framework (FIF) [11], students identify and categorize their knowledge gaps, formulate their research questions, and explore their findings. Students are taught these skills of information management and information mastery [12] early on in their pre-clerkship curriculum within the Health Systems Science (HSS) curricular thread. Incorporating these research presentations in PPPC met two needs in our curriculum: early incorporation of practice and application of emerging EBM skills, and meeting the LCME requirement for students to engage in regular self-directed learning [10].

A distinctive feature of PPPC is the active integration of the health sciences librarians [13]. Librarians provide individual feedback to students on the quality of the research questions they formulate, their search strategies, and the quality of the evidence they find.

Our study investigates our medical students’ abilities to apply the information mastery and EBM curriculum using this librarian feedback. In conjunction with our librarians and health systems science faculty, a standardized rubric was created to provide structured feedback to our students to assess their skills (Table 1).

**Table 1:** Standardized Assessment Rubric

Category	Excellent (4.0)	Good (3.0)	Fair (2.0)	Poor (1.0)
1. Was the research question relevant to this week's classes and patient?	Accurately identifies and prioritizes knowledge gaps, correctly categorizes and structures questions, and addresses educational needs of the team and/or the patient in the case.	Accurately identifies and prioritizes knowledge gaps and correctly categorizes and structures questions.	Identifies and prioritizes knowledge gaps but does not categorize and structure question to match identified gap.	Unable to articulate specific knowledge gap and/or inaccurately categorizes or structures questions.
2. Correctly categorized question using FIF	Critically evaluates the clinical question using the FIF and identifies background or foreground questions and identifies the resources to use.	Evaluates the research question using the FIF, but may not fully consider all aspects of quality and relevance. Selects mostly appropriate resources.	Demonstrates basic understanding of the FIF and which resources to use. Uses resource evaluation criteria but applies them inconsistently.	Struggles to evaluate the type of clinical question using the FIF.
3. Used appropriate information resources?	Synthesizes information from multiple sources to provide a comprehensive and nuanced answer to the clinical question or research problem. Clearly articulates the strengths and limitations of the evidence.	Synthesizes information from multiple sources, but may miss some key connections or nuances. Applies the evidence to the clinical scenario with some limitations.	Synthesizes information from a limited number of sources. Application of evidence to the clinical scenario is basic and may lack depth. Selection of resources may include some irrelevant or lower-quality items.	Struggles to find information from EBM resources to synthesize information and unable to locate information from multiple sources.
4. Clearly described search strategy or keywords?	Organizes and manages information effectively using appropriate tools and techniques. Uses advanced Search techniques MeSH terms and Boolean operators effectively.	Organizes and manages information adequately. Uses MeSH terms and filters but the search is not well structured.	Demonstrates basic information management skills with limited search refinement	Struggles to organize and manage information. Struggles to construct meaningful search
5. Were all materials properly cited?	Accurately cites all sources using a consistent and appropriate citation style including images.	Some materials were cited but not all. Cites most sources correctly, but may have minor errors in formatting or consistency.	Less than half of materials are cited. Lacks consistency in citing information resources used in presentation. Citation accuracy and consistency need improvement.	Struggles to understand the necessity of citing materials used in student presentations. Citations are incomplete, inaccurate, or inconsistent.
6. Did the student presentation mention Social Determinants of Health?	These questions are required and tracked in the rubric but were not assessed.			
7. Which Social Determinants of Health are mentioned?				

Given the early introduction of this curriculum and the ease with which 21st century students utilize technology, we anticipated that students can effectively locate resources and information, but had concerns over the quality of information resources used due to their reliance on google, AI search engines and other non-vetted sources [14].

First year medical students were introduced to the Patient Presentation Problem Based Learning Curriculum (PPPC) via a lecture during their medical school orientation and were given an example PowerPoint research presentation and a template. This template guides the creation of their presentations and includes the assessment components. Students learn information mastery in our longitudinal HSS curriculum in 6 distinct 2-hour sessions, starting within the first few weeks of medical school. The first of these sessions teaches students about the appropriate EBM information resources to use for PPPC presentations. The additional 5 information mastery sessions are given throughout the remainder of the pre-clerkship curriculum and are co-taught by librarians. These cover instruction on searching techniques, using FIF [11], asking PICO questions, and evaluating resources and information. Students begin applying these skills regularly in PPPC during their first weeks of medical school and create a presentation approximately once a month based on the weekly PPPC case. Students receive verbal feedback during class from faculty and peers, as well as written feedback from librarians and one peer reviewer.

There were 861 student presentations reviewed by our librarians for two cohorts of students from August 2022 through December 2023. The data showed that all presentations used the provided PPPC presentation template. Librarians reviewed student presentations from PPPC during the first 5 courses of the pre-clerkship curriculum, which span the first year of medical school. These courses included two foundational courses, Molecular and Cellular Principle (MCP), Structural Principles (SP) and three systems courses, Infection Immunity & Cancer (I2C), The Developing Human (TDH), and Homeostasis & Allostasis (HA).

The study was approved via the Hacken Longitudinal Outcomes of Medical Education (longMED) Hackensack Meridian Health IRB protocol number Pro2018-0308. All feedback data about student presentations was sent to the “honest broker”, a neutral third party who de-identifies the data and ensures that student information is stripped of direct identifiers, making it less likely that individuals can be identified. Students who opted out of longMED were not included in the study. Student's question categorization skills were assessed after learning about the FIF in a large-group classroom session held during the first weeks of medical school. A total of 595 presentations were assessed over the course of the pre-clinical curriculum.

We specifically looked at 5 components on the rubric that we felt best analyzed our students’ abilities in information mastery and self-directed learning. These included: appropriate scope of the question, is the question correctly categorized as a background or foreground question utilizing the FIF, appropriateness of information resources used, search strategy and correct citations using the American Medical Association (AMA) Manual of Style [15]. Statistical analysis was conducted using Stata 18 (Stata Corp, College Station Tx). Percentages of correct answers were calculated, and the trend of the percent correct over time was calculated using the Jonckheere's non parametric test. We also performed the Friedman test of differences across category since the high initial performance perhaps made detection of trend using Jonckheere's test inappropriate. All statistical tests were two sided and a p-value of 0.05 was considered statistically significant.

Results from the librarian review are demonstrated in Table 2. After being introduced to the PPPC curriculum, the introductory information mastery curriculum, and reviewing their expectations via a rubric, the majority of students (99.5%) were able to propose a research question with an appropriate scope. Most students (88.6%) were also able to incorporate the FIF into their presentation, describe their search strategy and keywords (95.5%), as well as find reliable sources via the FIF (94.5%). Nearly all of the students included a bibliography with proper citation (97%).

**Table 2:** Percentages of presentations that met the five studied rubric components.

	Appropriate Scope	Correctly Categorized Question	Appropriate Information Resources	Search Strategy	Accurate Citations
Yes	592 (99.5%)	527 (88.6%)	562 (94.5%)	568 (95.5%)	577 (97.0%)
No	3 (0.5%)	68 (11.4%)	33 (5.5%)	27 (4.5%)	18 (3.0%)

The test of trend over time using Jonckheere's non-parametric test was not statistically significant with a P value 0.13. Change over time is included in Table 3 and Figure 1. Testing using the Friedman test across the variables also did not reveal a statistically significant change over time.

**Table 3:** Change over time from the first pre-clerkship course (MCP) thru the fifth pre-clerkship course (HA). Acronyms stand for MCP (Molecular & Cellular Principles), SP (Structural Principles), I2C (Infection, Immunity, & Cancer), TDH (The Developing Human), HA (Homeostasis & Allostasis).

Course	Appropriate Scope	Correct Categorization	Appropriate resource use	Appropriate Search Strategy	Accurate citations
MCP	100%	78%	95%	92%	96%
SP	100%	86.7%	94.7%	95.6%	96.4%
I2C	96.7%	98.9%	94.6%	97.8%	97.8%
TDH	100%	91.3%	95.7%	97.8%	95.7%
HA	100%	95.3%	91.9%	94.2%	100%

The librarian response form had a section for free narrative response. The most common comment of feedback was related to the use of images to convey information. Other comments included suggestions on slide design and layout, as well as time management during presentations. The other most common comment was regarding the relevance and date of publication of resources used. Some examples of these narrative comments can be found in Table 4.

**Table 4:** Representative example of narrative comments from librarians.

Examples of Narrative Comments
I really like using the learning objectives so the group knows exactly what will be covered. Be aware of the publication dates of the articles you are using. The Nature article was published in 2000 which makes it 23 years old. The 2007 article makes the information older than 16 years! When using reference materials you try to keep the publication date no older than 5 years..to be sure it is current. Your presentation was well researched and very well organized. The CDC stats also were very useful. Nice job!
Beginning with definitions of dizziness is very useful so the group know exactly what you will be discussing. Be sure to cite any images on the slide on which they appear. You can just use a brief citation, where the slide was from and put the full citation on the last slide. Very useful to explore the History & Evaluation importance. Really well researched and well organized.
Starting your presentation with definition is a good way to be sure everyone knows exactly what you will be covering. The images you included really added to the content since they were all well labelled. Really well researched and organized in such a clear manner would be a good study tool for your group members. Nice job!
Be aware of the publication dates articles would are using as materials: An article published in 2010 is over 13 years old and dated. Look at article published in the last 5 years to be sure you are presenting the latest information. This was a really excellent presentation. Choosing a topic that discuss' social inequities and healthcare is so relevant in this case. Your presentation was very well researched and well organized. It was all tied to this weeks patient too.
This was really well done. The images really added to the content. It was well researched and well organized. Your group questions were thoughtful and I had the same questions about vaping and cardiovascular disease! Really well done and very relevant to the case of Mr. L.
Aesthetically lovely presentation. Looks clear and concise, but I worry about the scope of the presentation. Do you think you conveyed the proper amount of information in the time provided?

Librarians rated the scope of the clinical question as appropriate the majority of the time. However, comments suggested that particularly early in the curriculum, questions remained too broad to be answered effectively. Librarians would supplement the assessment rubric with a narrative to the student with suggestions to further focus the clinical background questions they are developing in early stages of PPPC. Examples of this can be found in Table 3. PPPC facilitators also gave feedback; however, we do not have this data as feedback was given verbally in real time. Because they are clinicians, PPPC facilitators may be better able to formulate narrower questions and thus give more focused feedback.

The appropriate categorization of the question had the most change over time from the first course to the last course assessed (MCP to HA) but overall scored the lowest across all skills assessed. The improvement likely occurred due to growing exposure and experience with the FIF [11]. At each of the information management and mastery teaching sessions, use of the FIF is reviewed. The overall percentage of presentations that appropriately categorized their questions was the lowest (<90%) than in any other category. Despite guidance to ask more background questions early in the pre-clerkship curriculum, students want to focus on clinical foreground questions about treatment of disease. However, due to limited content and medical knowledge, they may categorize their questions as foreground, but they are more likely to be background questions.

While we anticipated that students would utilize resources such as ChatGPT or Google, the majority of students utilized appropriate resources to find the answers to their clinical questions. This finding may be biased by the fact that students were aware that they would be assessed on resources used. It is possible that students used AI or Google in their initial search but were then able to reference appropriate resources. Furthermore, in our assessment, we did not distinguish between using evidence-based resources and patient-facing materials which may or may not be evidence-based.

Most students used an appropriate search strategy; however, this could be subject to the same bias as the previous category. Students again may have utilized Google or AI, but reported using search strategies that they knew evaluators were looking for. Citations were mostly done correctly, which is likely due to early and consistent exposure to free reference manager software (Zotero).

The students’ skills were strong and remained strong, which we feel was helped by the consistent reinforcement from librarian assessment. However, there may be other factors that contributed to strong student performance that were not captured by the rubric, such as prior knowledge, faculty support, or informal learning experiences.

Librarians were available and widely used, particularly early on in the curriculum, to assist students with preparing their research presentations. The curricular integration of the librarians is one of the major strengths of the evidence-based medicine and information mastery curriculum [13].

We evaluated presentations over the course of the first year of medical school, and we imagined that skills in information mastery and management would grow over time; however, our rubric did not change to assess advancing skills. Since performance was very high to begin with, there was a limited range for possible growth. Because of this, and due to limitations in librarian resources, the decision was made to only evaluate presentations during the first 12-months of the 16-month pre-clerkship curriculum. A future opportunity would be to modify the rubric as the students’ progress in their pre-clerkship curriculum to assess their growth, which could capture the evolution of their skills in the final four months prior to the start of clerkships. Additional skills in information management such as resource assessment or quality of evidence were not evaluated by our rubric. Utilizing these skills in the future may better assess how our students' skills develop over time.

We briefly reviewed the narrative comments from the librarians that were sent to the students with their feedback. While this was not a structured analysis of the content in the comments, common themes regarding the use of images to strengthen the presentation and the use of outdated articles did come up. Future studies could closely look at the narrative themes and their evolution over the course of the students' development through the pre-clerkship curriculum. Future research should look at assessment of these skills in students in the clinical learning environment. There is the potential for these skills to lapse as there are competing educational and clinical priorities; alternatively, these skills may be carried forward effectively into clerkships.

An additional benefit of this program was to enhance the student/librarian relationship. Students become very familiar with their librarians and are comfortable reaching out for assistance as they continue into their advanced years as medical students. Likewise, the relationship between the librarian and PPPC faculty has grown stronger as part of this collaboration; faculty get to know the librarians and see them as peers. A limitation of this program is the time commitment for librarians. The workload associated with assessment and feedback is significant and should be accounted for should other libraries implement a similar program as their institution.

## CONCLUSION

With early integration of librarians into an information mastery and information management curriculum, medical students participating in this program were able to successfully formulate clinical questions, correctly categorize them, and utilize appropriate resources to find evidence-based answers. The longitudinal integration of librarians into the PPPC program - where librarians provide weekly feedback to students for 12-months, reinforces the information management, mastery and EBM skills developed throughout the pre-clerkship curriculum. Narrative comments from the librarians were overwhelmingly positive, and particularly focused on the use of images to convey information. Comments also touched upon the use of outdated resources. Growth of the assessment rubric over time to meet the needs of students’ developing skills is necessary. Further studies can look at standardizing assessment of students’ EBM skills in the clerkship curriculum to see if these skills remain strong as students move from the classroom to the clinical learning environment, as competing interests in learning and clinical practice happen.

## Data Availability

Repository of data from reviewed research presentations are not publicly available as these are student assignments. All data generated and analyzed are included in this article. Further examples of librarian feedback are available from the corresponding author upon request.

## CLINICAL TRIAL REGISTRY

Not applicable, this is not a clinical trial.
